# Supplementary material for: A Global Screen for Assembly State Changes of the Mitotic Proteome by SEC-SWATH-MS
Source: Cell Syst. 2020 Feb 26;10(2):133–155.e6. doi: 10.1016/j.cels.2020.01.001 (PMC7042714; doi:10.1016/j.cels.2020.01.001)

Q9ULT8 | HECD1_HUMAN | HECTD1 KIAA1131
Monomer MW [kDa]: 289.384 Monomer expected elution fraction: 31

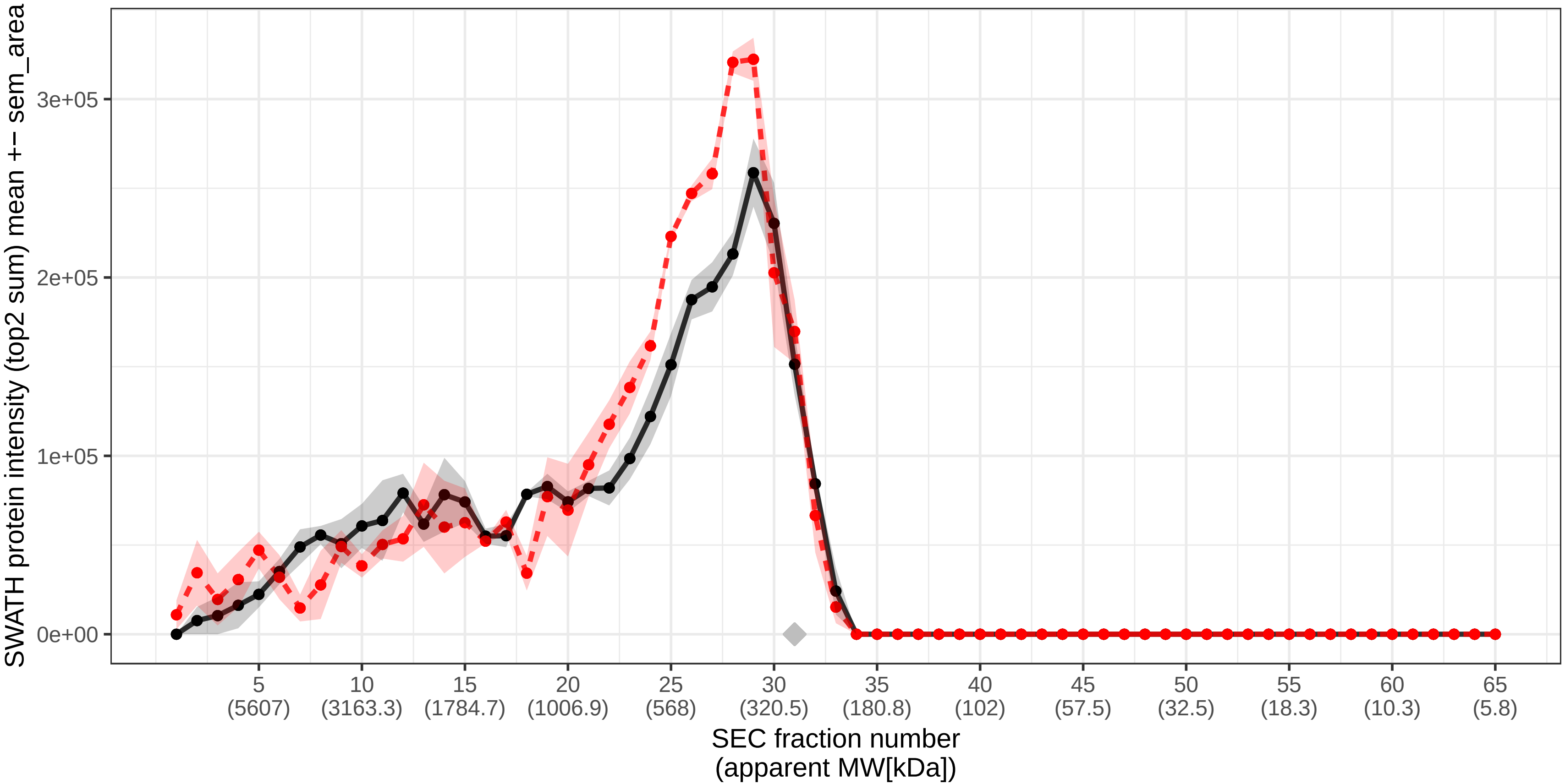

Supplement: Data S1. SEC-SWATH-MS Protein Chromatograms, Related to Figure 1 [file mmc6.zip › SECchrom_Q9ULT8_HECD1_HUMAN_HECTD1_KIAA1131.pdf]
